# Metformin Inhibits the Expression of Biomarkers of Fibrosis of EPCs In Vitro

**DOI:** 10.1155/2019/9019648

**Published:** 2019-03-18

**Authors:** Fei Han, Jie Shu, Shunjun Wang, Can-e Tang, Fanyan Luo

**Affiliations:** ^1^Department of Cardiothoracic Surgery, Xiangya Hospital, Central South University, Changsha, 410008 Hunan, China; ^2^The Institute of Medical Science Research, Xiangya Hospital, Central South University, Changsha, 410008 Hunan, China

## Abstract

Endothelial progenitor cells (EPCs) are a group of circulating cells with important functions in vascular repair and treatment of cardiovascular diseases. However, in patients with atrial fibrillation (AF), the number and function of EPCs reportedly are decreased. TGF-*β* is highly expressed in AF patients. In this study, we examined the effect of TGF-*β*1 on EPCs and the therapeutic outcome of metformin treatment on TGF-*β*1-induced EPCs. EPCs were induced with TGF-*β*1 at different concentrations (5 ng/ml, 10 ng/ml, and 20 ng/ml) for 48 h followed by western blot, qPCR, and immunofluorescence analyses to investigate changes in the levels of the fibrosis-related proteins, *α*-SMA, Col I, Col III, CTGF, and MMP-1. Live-dead cell staining was used to evaluate cell apoptosis. Compared with the control, TGF-*β*1 treatment significantly (*p* < 0.05) enhanced the levels of *α*-SMA, Col I, Col III, CTGF, and MMP-1 in a dose-dependent manner. The most effective concentration of TGF-*β*1 (20 ng/ml) was then used to induce fibrosis biomarker expression in EPCs, followed by treatment with metformin at different concentrations (0.5, 1, and 2 mmol/l). Metformin treatment suppressed TGF-*β*-induced expression of all above factors, with the effect at 2 mmol/l being significant (*p* < 0.05). Live-dead cell staining showed no difference among the control, TGF-*β*1-treated, and metformin-treated groups. In conclusion, our study showed that TGF-*β*1 induces the expression of fibrosis biomarkers in EPCs, which is attenuated by treatment with metformin. Thus, metformin may have therapeutic potential for improving EPC function in cardiovascular diseases.

## 1. Introduction

Endothelial progenitor cells (EPCs) are circulating cells that are considered to play roles in both vascular homeostasis and vasculogenesis [1]. EPCs can be collected from mononuclear cells, bone marrow, and cord blood. Current evidence suggests that EPCs express certain surface markers of endothelial-specific cells and show various endothelial characteristics [2]. Specific EPC markers are CD34, CD133, and vascular endothelial growth factor receptor-2 (VEGFR-2). There is a consensus that EPC subgroups are functionally heterogeneous and that their functions are determined by the expression of specific molecules. EPCs have been categorized into early and late EPCs. Early EPCs are characterized by angiogenesis and have a number of endothelial features. Late EPCs show diverse growth patterns and are therefore also termed outgrowth EPCs [3]. The clinical neovascularization function of EPCs remains to be explored. Moreover, the factors determining EPC differentiation *in vivo* and the mechanisms underlying EPC migration to the endothelium or extravascular tissue lesions remain unknown [4]. The functions and levels of EPCs have also been associated with heart function [5], providing novel targets for vascular repair and treatment of cardiovascular diseases. The levels of circulating EPCs reportedly are reduced in patients with persistent atrial fibrillation (AF) [6]. In addition, EPC proliferation, tube formation of mature endothelial cells, and paracrine effects of EPCs are also reduced in persistent AF [7].

Transforming growth factor-*β*1 (TGF-*β*1) is a crucial member of the TGF-*β* superfamily, which compromises over 40 proteins [8] characterized by a common sequence and structure. TGF-*β*1 is closely linked with several cardiovascular pathophysiologies, including cardiac hypertrophy, ventricular remodeling, valvular diseases, and cardiac fibrosis [9]. TGF-*β*1 contributes to myocardial fibrosis in patients with AF. It also induces fibrosis marker expression in other cells and tissues [10–12]. TGF-*β*1 knockout ameliorates diastolic dysfunction by attenuating myocardial fibrosis [13]. Patients with AF display high TGF-*β*1 expression in the myocardium, which may affect EPC function or viability and lead to fibrosis marker expression in circulating EPCs. However, no study has investigated this hypothesis.

Metformin, a biguanide compound, is an oral drug widely used to treat type 2 diabetes mellitus [14–16]. Metformin has not only antihyperglycemic properties but also cardioprotective effects [17, 18]. Therefore, nondiabetic patients may also benefit from its cardioprotective function. Previous studies have shown that metformin treatment resulted in an increase in circulating EPCs in type 2 diabetes patients [19]. However, the effect of metformin on EPCs under fibrotic circumstances remains largely unknown. In this study, we tested our hypotheses that TGF-*β*1 may induce fibrosis marker expression in EPCs and that this might be attenuated by metformin.

## 2. Materials and Methods

### 2.1. Materials

EPCs derived from human umbilical cord blood were purchased from Guangxiu Hospital (Changsha, Hunan, China). Recombinant human TGF-*β*1 protein was procured from Abcam (ab50036), and metformin was obtained from Solarbio (D9351).

### 2.2. Cell Culture and Treatments

EPCs were seeded in 12-well plates at 5 × 10^5^cells/well and were cultured in endothelial cell basal medium-2 (Lonza, USA) in a humidified 5% CO_2_ incubator at 37°C.

Fibrosis was induced by treating the EPCs with TGF-*β*1 for 48 h. To find the most effective concentration of TGF-*β*1, EPCs were treated with 0, 5, 10, or 20 ng/ml TGF-*β*1. After 48 h, protein and mRNA were extracted for western blotting and qPCR, respectively. Immunofluorescence staining was used to evaluate Col I expression in each treatment group, and live-dead cell staining was used to measure cell viability.

After treating EPCs with 20 ng/ml TGF-*β* for 48 h, the cells were treated with 0.5, 1, or 2 mmol/l metformin. Nontreated cells and cells treated with TGF-*β*1 alone were included as controls. After 24 h, protein and mRNA were extracted for western blotting and qPCR, respectively. Col I expression and cell viability were assessed as mentioned above.

### 2.3. Western Blot Analysis

Cells were lysed in RIPA lysis buffer (Beyotime, China), and the total protein concentrations were detected using the BCA Protein Assay Kit (TaKaRa, Japan). The proteins were separated by 10% SDS-PAGE and then transferred to PVDF membranes. All membranes were blocked with 5% BSA. The membranes were incubated with a rabbit polyclonal anti-human collagen type I antibody (ab34710; Abcam, dilution 1 : 4000), a rabbit polyclonal anti-human collagen type III antibody (ab7778; Abcam, dilution 1 : 4000), an anti-alpha muscle actin antibody (ab5694; Abcam, dilution 1 : 1000), or *β*-actin (BS13278, Bioworld, dilution 1 : 1500) at 4°C overnight. Subsequently, each membrane was incubated with the corresponding secondary antibodies at room temperature for 1 h. Finally, we used the enhanced chemiluminescence kit to observe protein bands in a ChemiDoc XRS Plus c (Bio-Rad, USA).

### 2.4. RT-qPCR Analysis

Total RNA was extracted from EPCs using TRIzol (TaKaRa, Japan) and was reverse-transcribed using a Bio-Rad iScript™ cDNA synthesis kit. Total RNA (500 ng) was subjected to 45 cycles of qPCR using all-in-one qPCR Mix (GeneCopoeia, USA) on the CFX96TM Real-Time PCR Detection System (Bio-Rad, USA). *GAPHD* was used as an internal reference for normalization. Primer sequences for expression analysis for the different genes were as follows: *α*-SMA: forward, 5′CGTTACTACTGCTGAGCGTGA3′, and reverse, 5′AACGTTCATTTCCGATGGTG3′; Col I: forward, 5′GTGCTACTGGTGCTGCCG3′, and reverse, 5′CACACCCTGGGGACCTTCAG3′; Col III: forward, 5′ACATGGATCAGGCCAGTGGA3′, and reverse, 5′TTCCCCAGTGTGTTTCGTGC3′; CTGF: forward 5′GTTTGGCCCAGACCCAACT3′, and reverse, 5′GGAACAGGCGCTCCACTCT3′; MMP-1: forward 5′GTGCCTGATGTGGCTCAGTT3′, and reverse, 5′TCTTGGCAAATCTGGCGTGT3′; and GAPDH: forward, 5′CAGGAGGCATTGCTGATGAT3′, and reverse, 5′GAAGGCTGGGGCTCATTT3′.

### 2.5. Immunofluorescence

EPCs were fixed in paraformaldehyde (4%) and then blocked in PBS with 0.5% Triton X-100 and 10% NGS for 1 h. The EPCs were incubated with an anti-Col I monoclonal antibody at 4°C overnight, followed by incubation with a fluorescein isothiocyanate-conjugated goat anti-rabbit IgG secondary antibody at 37°C for 1 h. The cells were washed to remove a secondary antibody and were then stained with DAPI for 15 min. The cells were washed with PBS and observed under a fluorescence microscope.

### 2.6. Live-Dead Cell Staining

Cells were washed twice with PBS and then treated with a mix of 5 *μ*l of 16 mM PI and 5 *μ*l of 4 mM calcein acetoxymethyl (KGAF001; KeyGEN BioTECH, China) in 10 ml of PBS. After a 30 min incubation at room temperature, the cells were observed under a fluorescence microscope.

### 2.7. Statistical Analysis

All data are presented as means ± SEMs. Means were compared by one-way ANOVA followed by the Bonferroni or LSD post hoc tests. We used the SPSS 19.0 software. *p* < 0.05 was considered significant.

## 3. Results

### 3.1. TGF-*β*1 Induces Fibrosis Marker Expression in EPCs

**Figure 1:**
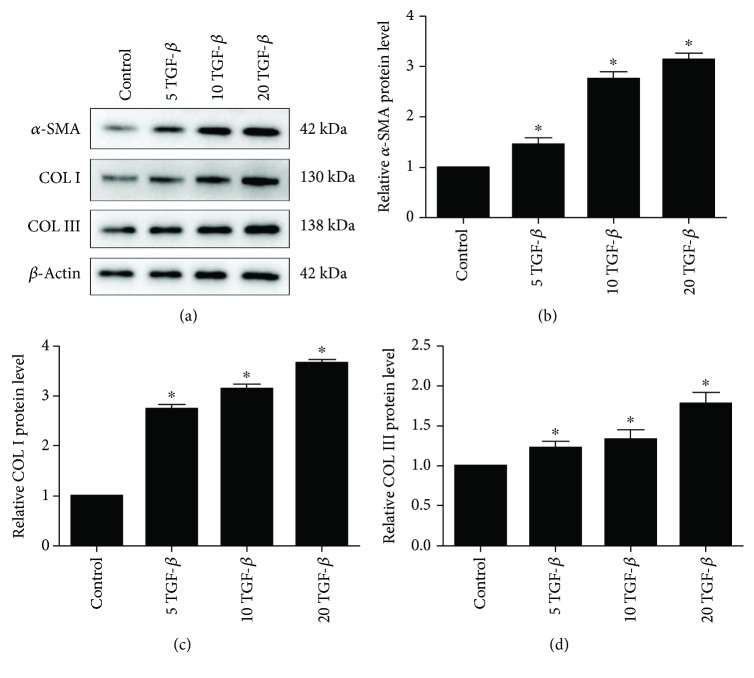
Relative protein expression of *α*-SMA, Col I, and Col III as determined by western blotting. Bars represent mean values ± SDs. *n* = 3. ^∗^*p* < 0.05.

**Figure 2:**
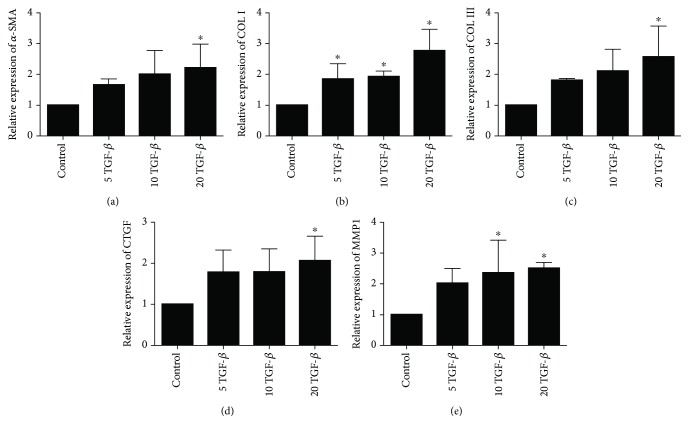
mRNA expression of *α*-SMA, Col I, Col III, CTGF, and MMP1 in EPCs as determined by RT-qPCR. ^∗^*p* < 0.05.

TGF-*β*1 is commonly used to induce fibrosis. To investigate whether TGF-*β*1 affects fibrosis marker expression in EPCs, we treated these cells with 5, 10, or 20 ng/ml TGF-*β*1 for 48 h. The protein levels of *α*-SMA, Col I, Col III, and some markers of fibrosis were assessed by western blotting. The levels of *α*-SMA, Col I, and Col III increased with increasing concentrations of TGF-*β*1 (*p* < 0.05 at all concentrations; Figure 1). Moreover, compared with Col III, *α*-SMA and Col I were increased more significantly. The mRNA expression of *α*-SMA, Col I, Col III, CTGF, and MMP1, detected by qPCR, increased in all the TGF-*β*1 groups, particularly in the 20 ng/ml group, and showed significant differences compared with the control group (*p* < 0.05; Figure 2).

**Figure 3:**
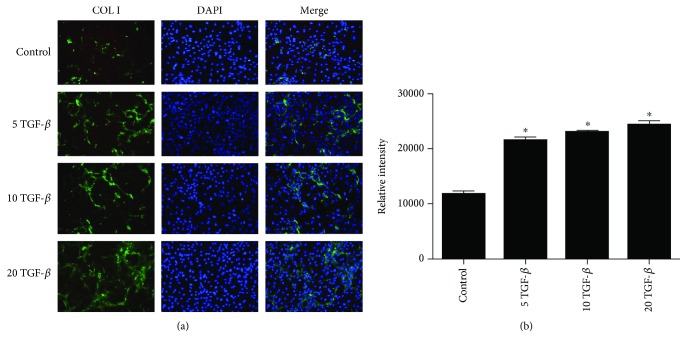
Immunofluorescence staining for Col I in EPCs treated with 0, 5, 10, or 20 ng/ml TGF-*β*1. ^∗^*p* < 0.05.

**Figure 4:**
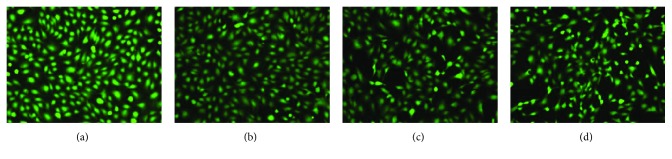
Live-dead cell staining: (a) control group and (b–d) 5, 10, and 20 ng/ml TGF-*β*1 treatment, respectively. No obvious apoptosis of EPCs was observed in each group. However, there were some cellular morphological changes in (c, d), and some stellate and flat cells were observed in these groups.

Immunofluorescence was used to visualize Col I in EPCs at 48 h after TGF-*β*1 treatment. Similar to the results of the western blot analysis, the relative intensities in the 5 ng/ml TGF-*β*1 group (21478.67 ± 617.14), 10 ng/ml TGF-*β*1 group (23009.00 ± 291.18), and 20 ng/ml TGF-*β*1 group (24277.00 ± 788.25) were significantly higher than those in the control group (11762.00 ± 578.09) (*p* < 0.05 at all concentrations; Figure 3). Using live-dead cell staining, we confirmed that there was no obvious apoptosis of EPCs in each treatment group, and no significant differences were found between the groups (Figure 4).

Together, these results indicated that TGF-*β*1 can indeed induce the expression of fibrosis biomarkers in EPCs, and 20 ng/ml is an appropriate concentration of TGF-*β*1 for induction.

### 3.2. Metformin Attenuates TGF-*β*1-Induced High Fibrosis Marker Expression in EPCs

**Figure 5:**
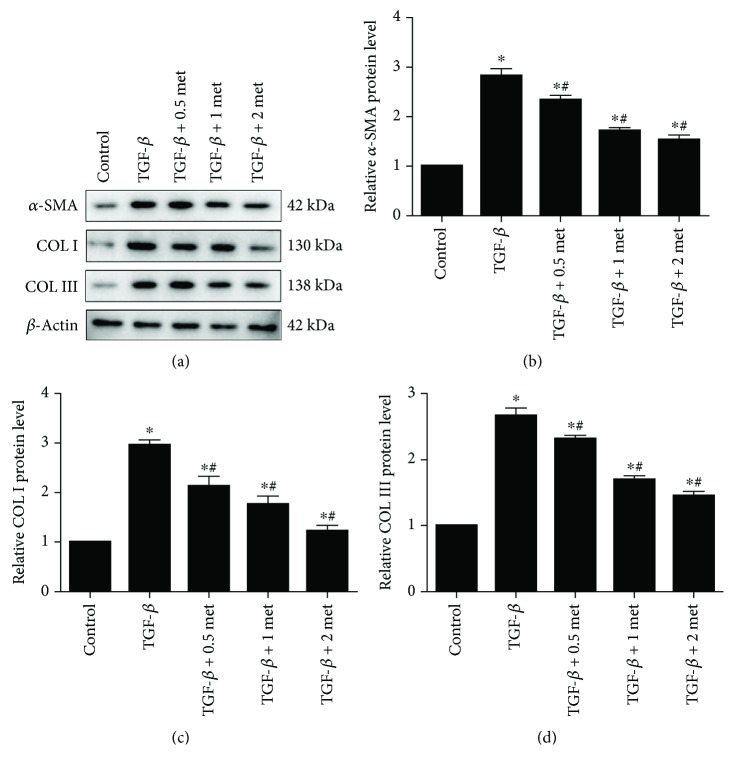
Relative protein levels of *α*-SMA, Col I, and Col III as determined by western blotting. Bars represent mean values ± SDs. *n* = 3. ^∗^*p* < 0.05 vs. the control group. ^#^*p* < 0.05 vs. the TGF-*β*1 group. met: metformin.

**Figure 6:**
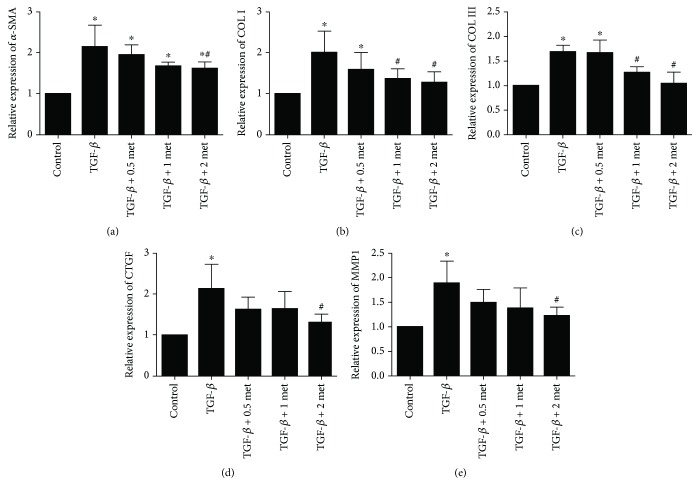
mRNA expression of *α*-SMA, collagen I, collagen III, CTGF, and MMP1 in EPCs as determined by RT-qPCR. ^∗^*p* < 0.05 vs. the control group. ^#^*p* < 0.05 vs. the TGF-*β*1 group. met: metformin.

Based on the above results, we induced fibrosis in EPCs by exposing them to 20 ng/ml TGF-*β*1 for 24 h and then treated the cells with 0.5, 1, or 2 mmol/l metformin. Western blot analysis indicated that the levels of *α*-SMA, Col I, and Col III in the TGF-*β*1 groups treated with metformin were significantly lower than those in the group treated with TGF-*β*1 alone (*p* < 0.05; Figure 5), but these were still above those in the control group (*p* < 0.05; Figure 5). The effect of metformin was dose-dependent. These results were further validated by RT-qPCR, which also showed decreasing mRNA levels of *α*-SMA, Col I, Col III, CTGF, and MMP with increasing concentrations of metformin (Figure 6). The 2 mmol/l metformin group showed the most significant difference compared with the TGF-*β*1 alone group (*p* < 0.05; Figure 6).

**Figure 7:**
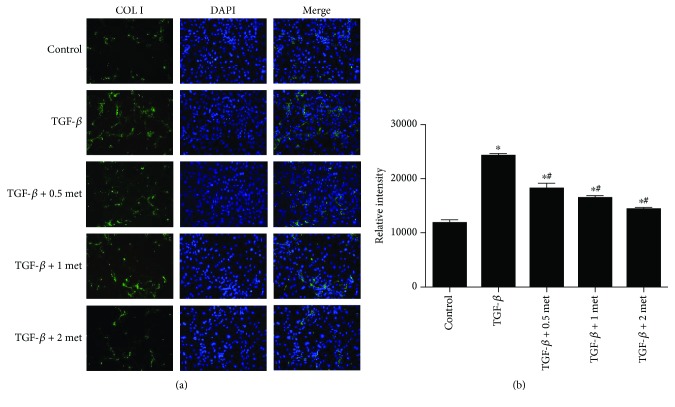
Immunofluorescence staining for Col I in cells treated with 20 ng TGF-*β*1 and 0.5, 1, and 2 mmol/l metformin. Nontreated cells and cells treated with TGF-*β*1 alone were included as a control. ^∗^*p* < 0.05 vs. the control group. ^#^*p* < 0.05 vs. the TGF-*β*1 group. met: metformin.

**Figure 8:**
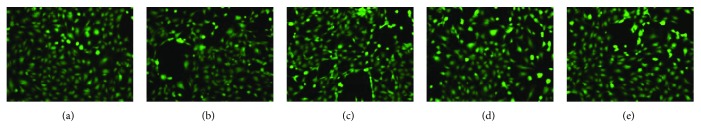
Live-dead cell staining: (a) control; (b): 20 ng TGF-*β*1; (c) TGF-*β*1+0.5 mM metformin; (d) TGF-*β*1+1 mM metformin; (e) TGF-*β*1+2 mM metformin. No obvious apoptosis of EPCs was observed in any group. However, there were some cellular morphological changes in (b) to (e); some stellate and flat cells were observed in these groups.

Immunofluorescence analysis of Col I showed a significant reduction in fluorescence intensity in the metformin-treated cells when compared with cells treated with TGF-*β*1 alone (Figure 7). The relative intensities in the 0.5 mmol/l (18174.33 ± 962.40), 1 mmol/l (16427.33 ± 412.02), and 2 mmol/l (14337.00 ± 357.36) metformin groups were lower than those in the TGF-*β*1 alone group (24248.00 ± 401.66). In addition, the relative intensities in all of these groups were higher than those in the control group (11781.33 ± 603.43) (*p* < 0.05). Thus, metformin could inhibit TGF-*β*1-induced fibrosis marker expression to a certain extent (Figure 7). Live-dead cell staining showed that there was no obvious apoptosis of EPCs in any of the groups (Figure 8).

Together, the results indicated that metformin could attenuate TGF-*β*-induced high expression of fibrosis biomarkers in EPCs, and 2 mmol/l metformin had the strongest effect among the concentrations tested in the current study.

## 4. Discussion

In the present study, in order to find the most appropriate concentrations of TGF-*β*1 and metformin, we applied some different concentrations according to the literature review. 5 ng/ml [20], 7 ng/ml [21], and 10 ng/ml [22, 23] TGF-*β*1 were used, respectively, to induce fibrosis of different cells in previous studies. Another study showed that in patients with atrial fibrillation, the serum levels of TGF-*β*1 were ranged from 15.4 to 27.6 ng/ml [24]. So, we applied three concentrations of TGF-*β*1 (5, 10, and 20 ng/ml) to induce fibrosis biomarker expression in EPCs, and we found that 20 ng/ml TGF-*β*1 had the most significant fibrosis-inducing effect. The live-dead cell staining assay indicated that although TGF-*β* induced fibrosis in EPCs, it did not cause apoptosis of the cells. Thus, the reduction in circulating EPCs in patients with permanent AF may be an outcome of fibrosis rather than of apoptosis. Therefore, protecting the function of EPCs might be an important therapeutic strategy in these patients. According to previous work, 0.5 mmol/l [21] and 1 mmol/l [20, 21, 25] metformin were used to treat cardiac, liver, or lung fibrosis, respectively. In our study, we tested the effects of metformin at three concentrations (0.5, 1, and 2 mmol/l), and we found that 2 mmol/l metformin attenuated fibrotic expression in EPCs the most effectively, as evidenced by downregulated mRNA and protein expression of Col I, Col III, and *α*-SMA. Thus, metformin inhibited cellular fibrosis in a dose-dependent manner, without having any cytotoxic effect on the cells.

EPCs were first described as a major contributor to cardiovascular tissue regeneration [4]. EPCs can develop into differentiated endothelial cells. In addition, they can develop into cardiomyocytes, smooth muscle cells, and skeletal muscle cells. Miyata et al. [26] confirmed that the bone marrow-derived EPC cell line, TR-BME2, differentiated into cells with a smooth muscle phenotype in the presence of PDGF-BB. Endothelial repair and neovascularization of ischemic organs are seemingly related to EPCs. The clinical neovascularization function of EPCs remains to be explored. Moreover, the stimulating or inhibiting factors affecting EPC differentiation *in vivo* and the underlying mechanisms of EPC migration to the endothelium or extravascular tissue lesions remain unclear [27]. Intriguingly, studies have shown that the population of EPCs greatly expands in the first stage of acute myocardial infarction, showing that EPCs may be beneficial for regeneration [28]. Despite decades of efforts, there is a long way to go before stem cell-based therapies are able to cure cardiac diseases. In this study, we reasoned that circulating EPCs might interact with and be influenced by the vascular microenvironment, which includes factors such as TGF-*β*, which would lead to their dysfunction. Clinically, the higher risk of cardiovascular diseases stems from a decrease in the level of EPCs. The underlying mechanisms include oxidative stress and increased nitric oxide activity, which may impair EPC activation and mobilization. Further, EPC exhaustion may be caused by continuous endothelial dysfunction [2]. Taken together, these findings indicate that EPCs play a significant, undeniable role in cardiovascular biology. In fact, the distribution and numbers of circulating EPCs reflect the cardiovascular condition.

Fibrosis can be present in various organs and tissues, including the heart, liver, lungs, and kidneys. In the process of fibrosis, the tissue can express high-level fibrogenic markers [22], such as *α*-SMA, Col I, Col III, CTGF, and MMP1. In addition, in some fibrotic tissues, TGF-*β*1 expression tends to increase. For example, most AF patients present atrial fibrosis, and TGF-*β*1 expression is higher in the atrial tissues than in the ventricles of AF patients [29]. In turn, high-level TGF-*β*1 aggravates AF, and TGF-*β* is often used as an effective agent for inducing experimental tissue and cell fibrosis [23]. TGF-*β* can induce fibrosis in different cell types, including cardiomyocytes [22], pulmonary interstitial cells [30], renal cells [31], and liver cells [32]. TGF-*β* greatly promotes collagen synthesis and accumulation [33], which was also observed in the current study. TGF-*β* signals and cellular effects are transmitted into inner cells by transcription factors called Smads, which are closely linked with fibrosis-associated diseases in mammals [34]. Canonical TGF-*β* signaling mobilizes Smad2 and Smad3, which control fibrosis by promoting gene expression, and TGF-*β*-Smad2/3 signaling in activated tissue-resident cardiac fibroblasts is a principal mediator of the fibrotic response [22]. Smad3 underlies the proper induction of TGF-*β*-responsive genes, such as Acta2, Col I, Col III, CTGF, and fibronectin [35]. *α*-SMA, Col I, Col III, CTGF, and MMP1 are important downstream effectors of the profibrotic actions of TGF-*β*1 [35–37]. TGF-*β*1 knockout could ameliorate myocardial fibrosis [13]. However, TGF-*β* regulates cell proliferation, differentiation, and apoptosis, and TGF-*β*-related proteins have key roles in development, tissue homeostasis, and disease [38]. TGF-*β* reportedly induces growth of cultured neonatal cardiac myocytes and plays a central role in the repair of many tissues after injury. It has been confirmed that exogenous TGF-*β* stabilizes the beating rate of neonatal rat cardiac myocytes cultured on fibroblast matrix and sustains their spontaneous rhythmic beating in serum-free medium [39]. Goumans and Dijke [40] argued that TGF-*β* has a pivotal role in endothelial and smooth muscle cell proliferation, differentiation, migration, tube formation, and sprouting. Thus, blocking TGF-*β* might affect the above functions.

Metformin is widely used to treat type 2 diabetes. The fact that metformin has cardiovascular protective functions beyond its antihyperglycemic effects has been confirmed by the United Kingdom Prospective Diabetes Study [41, 42]. Increasing evidence suggests that metformin also has a cardioprotective function [43]. Cittadini et al. [44] suggested that metformin could attenuate left ventricular remodeling. Further, metformin improves systolic and diastolic function along with pump efficiency. Its pharmacological action is mediated through the activation of AMP-activated protein kinase (AMPK), which regulates not only energy homeostasis but also responses to stress, including reactive oxygen species (ROS) stress. ROS have been implied in TGF-*β*-induced myofibroblast differentiation [25]. Yu et al. [45] suggested that metformin improved angiogenesis of EPCs in diabetic mice via activation of the AMPK/eNOS signaling pathway. Metformin reportedly attenuates cardiac fibrosis by inhibiting TGF *β*1-Smad3 signaling [20]. Goto et al. [21] reported that metformin could inhibit liver fibrosis through SNF1/AMP kinase-related kinase-mediated enhancement of TGF-*β* signaling. It has been reported that metformin can mitigate carbon tetrachloride-induced TGF-*β*1/Smad3 signaling and liver fibrosis in vivo [34]. Previous studies in vitro confirmed that metformin can inhibit TGF-*β*1-induced inflammatory and fibrotic responses through Smad3, ERK1/2, and P38 pathways in human renal proximal tubular cells [46].

In summary, metformin can inhibit the fibrotic process in some organs and tissues. The present study explored the effects of TGF-*β*1 and metformin on EPCs. The findings suggested that metformin attenuates TGF-*β*1-induced fibrosis marker expression and collagen accumulation in EPCs. This may allow EPCs to maintain a better cellular state and reduce the requirement for EPCs to repair damaged parts, such as vessels and myocardium. This study is limited by the fact that we did not explore whether metformin could attenuate fibrosis of EPCs in vivo; animal experiments need to be conducted to confirm our results. Additionally, while this study elucidates changes in fibrotic biomarkers caused by TGF-*β*1 and metformin, the molecular mechanisms underlying the antifibrotic effect of metformin remain unexplored.

## 5. Conclusion

TGF-*β*1 can induce fibrotic biomarker expression in EPCs, and metformin can attenuate this progress.

## Data Availability

The data used to support the findings of this study are included within the article.
